# Comparison of the safety and efficacy of unilateral biportal endoscopic lumbar interbody fusion and uniportal endoscopic lumbar interbody fusion: a 1-year follow-up

**DOI:** 10.1186/s13018-022-03249-4

**Published:** 2022-07-23

**Authors:** Y. Z. Xie, Y. Shi, Q. Zhou, C. Q. Feng, Y. Zhou, T. Li, Y. Yu, X. H. Fan

**Affiliations:** 1 Hospital of Chengdu University of Traditional Chinese Medicine, No. 39 shi-er-qiao Road, Chengdu, 610072 Sichuan Province People’s Republic of China; 2 Chengdu University of Traditional Chinese Medicine, No. 1166 Liu-tai Avenue, Chengdu, 611137 Sichuan Province People’s Republic of China

**Keywords:** Unilateral biportal endoscopic lumbar interbody fusion, Uniportal endoscopic lumbar interbody fusion, Surgical outcomes

## Abstract

**Objectives:**

To compare the short-term outcomes of unilateral biportal endoscopic lumbar interbody fusion (BLIF) and uniportal endoscopic lumbar interbody fusion (ULIF).

**Methods:**

Sixty patients diagnosed with L4/5 spinal stenosis who underwent BLIF and ULIF were included (30 in each group). Clinical evaluation was performed preoperatively and postoperatively in the 1st week, 1st month, and 1st year. Factors such as the visual analogue score (VAS), Oswestry Disability Index (ODI), operative time, surgical complications, and radiological outcomes (fusion rate, screw loosening, and cage subsidence) were compared between the two groups.

**Results:**

All patients showed improved mean VAS and ODI at all three postoperative follow-ups, and no statistically significant differences were detected between the BLIF and ULIF groups. The mean operative time in the BLIF group was shorter than that in the ULIF group. Nerve root injury occurred in two patients in the BLIF group, while leakage of cerebrospinal fluid occurred in one patient in the ULIF group. All adverse events were treated adequately prior to discharge. The fusion rates with definite and probable grades were significantly higher in the BLIF group than that in the ULIF group. One case of cage subsidence with no screw loosening occurred in each group.

**Conclusion:**

Both BLIF and ULIF are safe and effective surgical techniques. Compared with ULIF, BLIF has the advantages of shorter operative time and a higher fusion rate. Other merits of BLIF include a wider surgical field, greater maneuverability of instruments, visibility during cage implantation, and transverse orientation of the cage.

## Introduction

Minimally invasive spinal surgery is currently flourishing worldwide with the advent of several novel and practical lumbar techniques coming forth constantly, such as channel-assisted fusion operation, microendoscopic discectomy, microscopic and extreme/direct lateral interbody fusion, oblique lateral interbody fusion, and percutaneous endoscopy [1–3]. Given the advantages of a clear operative field, lesser trauma, and rapid recovery, percutaneous lumbar endoscopy has obtained widespread approval [4, 5]. Upon its introduction, the technique was purely aimed at treating disk herniation, but its indications have expanded from pure lumbar disk herniation to complex spinal stenosis [6, 7]. In the past recent several decades, uniportal percutaneous lumbar endoscopy is the mainstream technique for coping with purely disk herniation and decompression without fusion. Particularly for Transforaminal Endoscopic Spine System (TESSYS) and Yeung Endoscopic Spine Surgery (YESS) techniques, the most two popular schools of uniportal percutaneous lumbar endoscopy operate within the Kambin triangle [8, 9]. Its definite curative effect has been reported much in the literature [4, 10, 11]. However, biportal percutaneous lumbar endoscopy has a longer history. In 1980s, Kambin primarily used arthroscope to perform discectomy [12]. Till 1996, the technique has been steadily ameliorated by several scholars to treat sorts of lumbar diseases such as lumbar infection and spinal canal stenosis [13–16]. Yet, as TESSYS and YESS techniques have been advanced as a milestone in the developmental process of percutaneous lumbar endoscopy, biportal technique was hammered and has faded out of the mainstream of academia with a few surgeons still insisting.

Currently, researchers in this field have begun to explore the possibility of fusion under endoscopic guidance, since primary discectomy is too simple to address complicated issues including instability, spondylolisthesis, and severe lumbar degenerative disease combined with low back and lower limb pain [17, 18]. Surprisingly, biportal percutaneous lumbar endoscopy revives due to its broader surgical field and is put on a par with uniportal percutaneous lumbar endoscopy. Nevertheless, the pros and cons of these two techniques have never been reported by the literature so far and the option of these two techniques is short of reference standard. Hence, endoscopic lumbar interbody fusion merits further exploration.

**Fig. 1:**
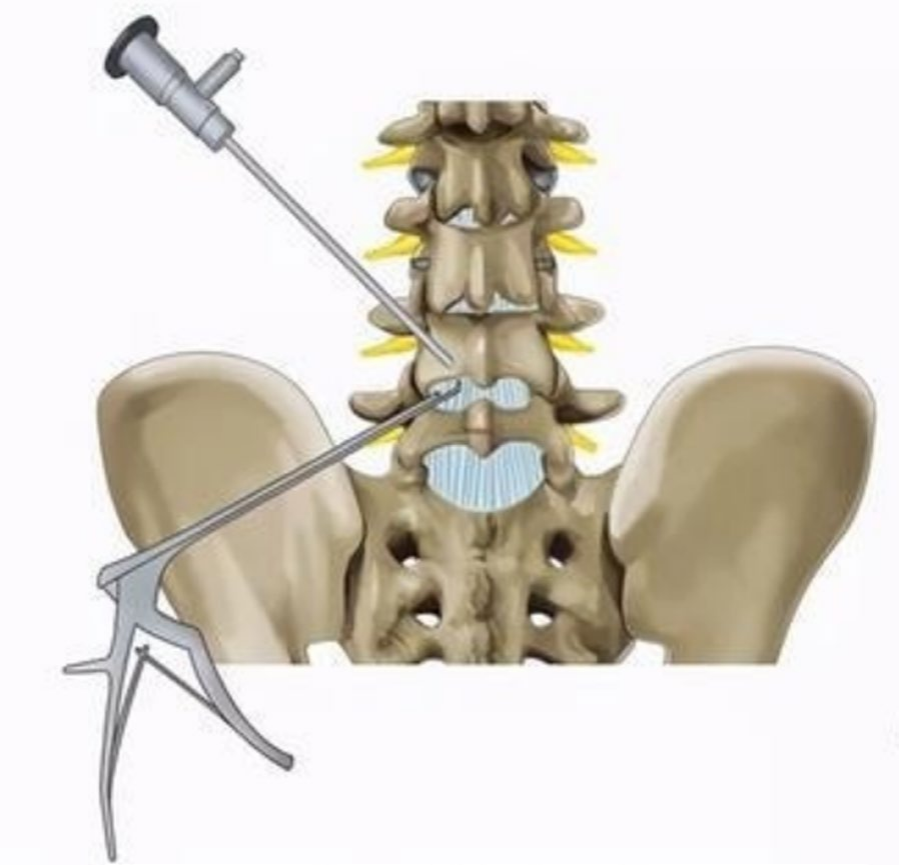
Unilateral biportal endoscopic lumbar interbody fusion (BLIF)

**Fig. 2:**
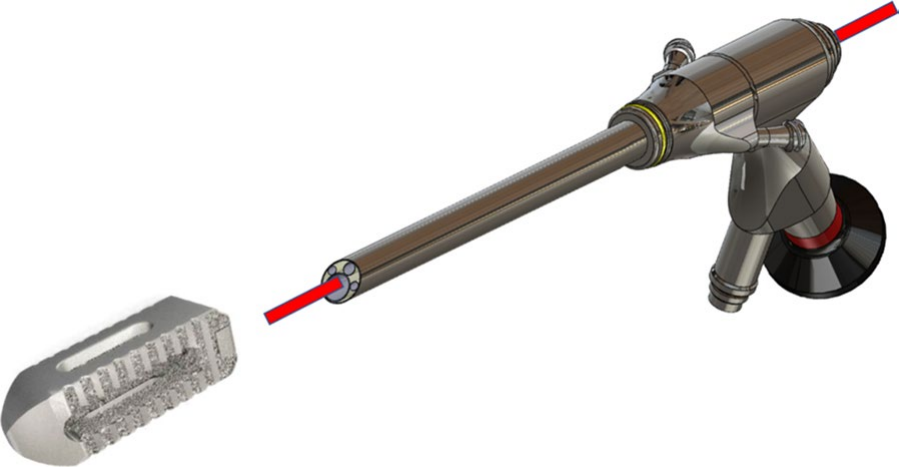
Uniportal endoscopic lumbar interbody fusion (ULIF)

The author’s institution, the Hospital of Chengdu University of Traditional Chinese Medicine, is a research center located in the western region of China. The surgical team here has always been committed to the development of lumbar percutaneous endoscopy; as for the endoscopic application, unilateral biportal endoscopic lumbar interbody fusion (BLIF) (Fig. 1) and uniportal endoscopic lumbar interbody fusion (ULIF) (Fig. 2) are both frequently used techniques in our practice. In this retrospective study, we aimed to compare and contrast the safety and efficacy of these two techniques.

## Methods

### Study participants

A total of sixty patients diagnosed with L4/5 spinal stenosis, who underwent BLIF and ULIF at the Hospital of Chengdu University of Traditional Chinese Medicine from 1 July 2019 to 1 July 2020, were included and classified into BLIF and ULIF groups, respectively. The option of BLIF or ULIF for a patient was based on whether a bilateral decompression was necessarily needed. If so, BLIF would be chose. Otherwise, ULIF would be picked. The BLIF group (*n* = 30) included 17 men and 13 women, aged 40–60 years old, with an average age of 49.1 years. The ULIF group (*n* = 30) included 16 men and 14 women, aged 42–60 years old, with an average of 51.2 years. Preoperative symptoms and signs were correlated to the imaging data, and the responsible segment was identified as L4/5 in all concluded patients. Alongside imaging data, we also used several relative indicators to analyze each group, including the visual analogue scale (VAS), the Oswestry Disability Index (ODI), operative time and fluoroscopy time. All surgeries were performed by the same surgeon and his team, having at least 10 years of experience in endoscopic spinal surgeries.

### Ethical considerations

The study was reviewed by the ethics committee of the affiliated Hospital of Chengdu University of Traditional Chinese Medicine (approval no.NT-6656). Informed consent was taken from all patients for their participation in the study.

### Diagnostic criteria

The diagnostic criteria used for lumbar spinal stenosis were as follows: [19]

1. Neurological examination (including the straight leg raising test).

2. Magnetic resonance imaging (MRI)/computed tomography (CT) scans.

3. Observation of gait.

The following three additional tests were performed to rule out other conditions and/or diseases:

1. Examination of foot pulses and estimation of ankle brachial index (ABI).
2. Examination of the hip.
3. Assessment of cervical myelopathy.

Functional X-ray imaging was not used for diagnosis as it was considered useful only for surgical decision making.

### Inclusion criteria

Patients were enrolled if they met the following inclusion criteria: 1. L4/5 spinal stenosis detected by thorough examination of lumbar CT and MRI films; 2. Age ≥ 20 years or ≤ 60 years; 3. Signs and symptoms of L4/L5 spinal stenosis as confirmed by CT/MRI imaging; 4. Persistence of clinical symptoms despite conservative treatment for more than 3 months, or disease relapse with more severe symptoms after an initial period of improvement; and 5. No evidence of contraindication to surgery.

### Exclusion criteria

Patients were excluded when: 1. The patient met the criteria for enrollment, but was receiving another treatment; 2. Patients with multi-segmental lumbar spinal stenosis or additional spinal disorders such as a tumor, fracture, or idiopathic scoliosis; and 3. Prior relevant surgical history.

### Surgical equipment

1. Biportal Endoscopic Lumbar Intervertebral Fusion System (VantageTM, Taoyuan City 320, Taiwan, China)
2. Percutaneous Lumbar Endoscopy Instrument-series of PLUS (Joimax® GmbH, Karlsruhe, Germany)

### Surgical procedure

#### Surgical preparation

After obtaining a detailed medical history, a careful physical examination was performed. Relevant investigations including routine blood tests and coagulation function tests were performed. If the patient’s blood pressure or sugars were not well controlled, the surgery was withheld until they were regulated within the tolerable operating range. Preoperative CT and MRI scans of the lumbar spine were obtained as per requirements. If the patient had stents or metallic foreign bodies that precluded MRI examination, an electronic CT was performed. The diagnosis was reconfirmed after the examination results were obtained. Finally, VAS and ODI were performed before the surgery.

#### Intraoperative details

**Fig. 3:**
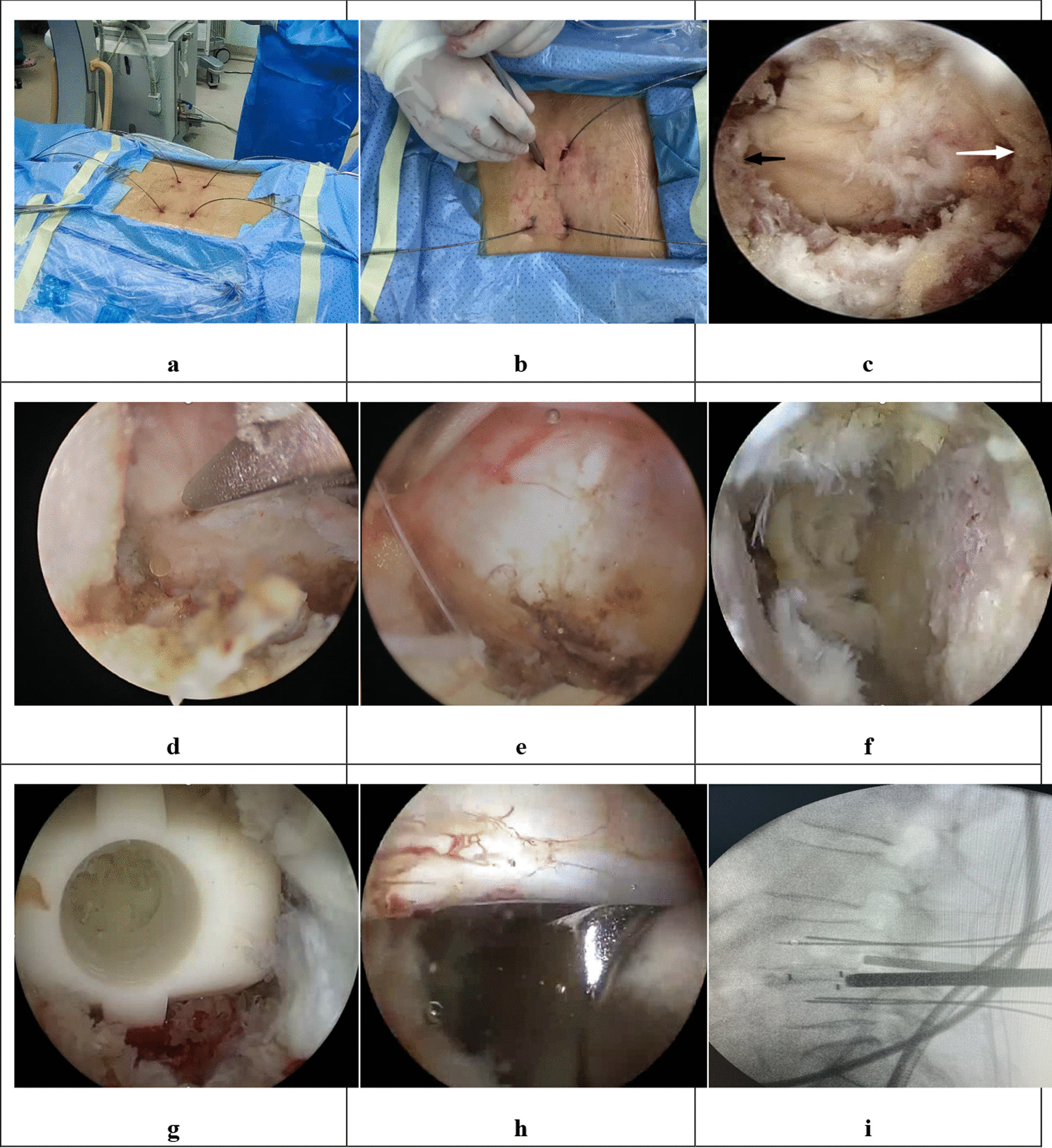
The combined figures show the steps of BLIF. **a** Guide wires’ insertion. **b** Soft tissue detachment. **c** Exposure of the superior margin of the L5 lamina (black arrow), interlaminar space and inferior edge of L4 lamina (white arrow).** d** Laminotomy. **e** Preparation of the bone graft bed with an endoscopic cobber. **f** Observation of bony bleeding sites. **g** The cage is seen through the endoscope. **h** The cage is justified in a transverse orientation with an impactor. **i** The correct location is confirmed under C-arm guidance

BLIF: After induction of general anesthesia, the patient was placed in the prone position. The surface markings of the pedicles, the puncture point, and the L4/5 intervertebral space were made, and the surgical area was routinely disinfected and draped. First, four pedicle screw-guide wires were inserted under intraoperative fluoroscopic guidance. Second, the endoscopic puncture needle was inserted into the point marked earlier. The C-arm confirmed that the needle had reached the laminar space. The intersection point of the laminar space and the puncture needle line on the operative side was used as a midpoint, and two 1 cm incisions at the cephalic and caudal sides were made about 1 cm from the midpoint. Then, the detacher was used to detach the soft tissue surrounding the interlaminar space. The working channel with the lens was inserted along the tube, and connected to the light source, the imaging system, and the water conduit. Third, soft tissue was cleared using an endoscopic grasper and radiofrequency ablation electrode to expose the superior margin of the L5 lamina, interlaminar space, and inferior edge of the L4 lamina. The intersection of the medial fringe of the L5 superior articular process and the L5 lamina superior border, an important anatomical marker called the point L, could be visualized through the endoscope. Next, the osteotome was used to remove a part of the lamina and facet joint. The Kerrison rongeur was also used to improve the precision of the laminectomy. Once the yellow ligament came into view, it was removed step by step to expose the dural sac and nerve root. After pulling the nerve using a nerve retractor, the nucleus pulposus was revealed and was resected using a grasper. After the disk was removed, the intervertebral space was cleared. The cartilage tissue adhering to the superior and inferior end plates was scraped with a curette and reamer, and bony bleeding sites on the endplate were observed. Endoscopic bone grafting was performed, and an appropriately sized cage was implanted under endoscopic guidance. Then, the cage was justified in a transverse orientation using an impactor. Fourth, we confirmed once more that the nerve root and dural sac were unwound without any nucleus pulposus remaining. After exiting endoscopy, four pedicle screws and two connecting rods were implanted percutaneously. Finally, the surgical site was rinsed thoroughly with a large volume of saline, and the surgical incision was sutured after ensuring hemostasis. Ropivacaine and tranexamic acid injection were injected into the wound before covering it with a sterile dressing (Fig. 3).

**Fig. 4:**
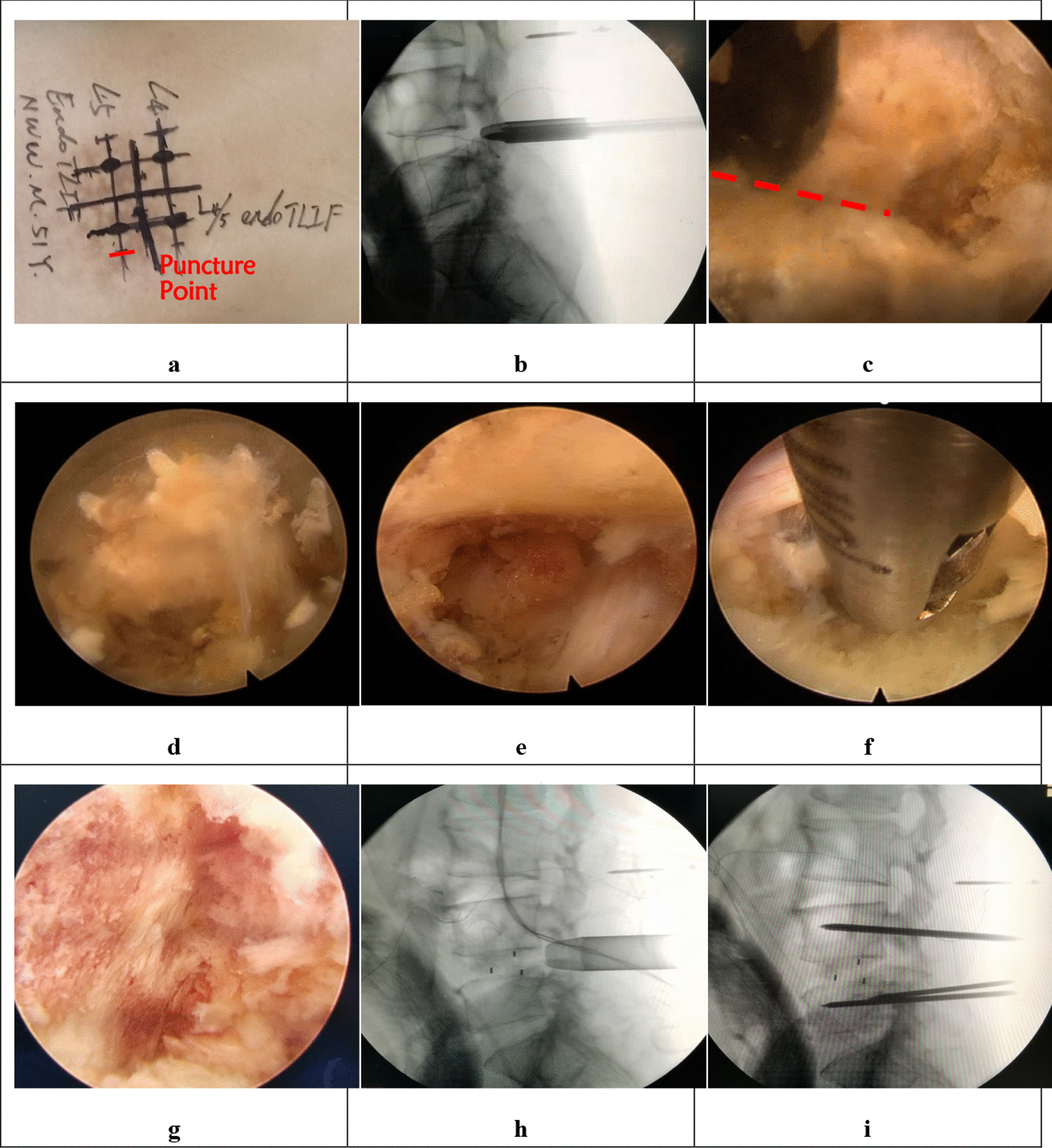
The combined figures show the steps of ULIF. **a** Operative marking. **b** The working channel is inserted. **c** Facet joint is revealed by clearing the soft tissue surrounding.** d** Laminoplasty by using an endoscopic rimmer. **e** The disk is revealed by rotating the working channel after foraminoplasty. **f** The endoscopic grasper was used to perform discectomy. **g** The bed for bone graft is well prepared. **h** The location of the cage is ensured under C-arm guidance. **i** The guide wires are inserted to prepare for the insertion of pedicle screws

ULIF: After general anesthesia, the patient was placed in the prone position, and the C-arm was positioned so as to locate the horizontal line of the L4/5 laminar space. The puncture point was situated on the lateral side, about 1 cm from the posterior midline. Then, we marked four surface projections for the pedicles, one of which was both the insertion point of the pedicle screw and the puncture point of endoscopy. The surgical area was routinely disinfected and draped. First, an incision was made using a scalpel and an endoscopic primary dilating tube was put in. Through C-arm fluoroscopy, we confirmed that the tube reached the interlaminar space. Next, the advanced dilating tube was placed through the primary tube. The working channel with the lens was inserted along the tube, connected to the light source, the imaging system, and the water conduit. Third, soft tissue was cleared using an endoscopic grasper and radiofrequency ablation electrode to expose the superior edge of the L5 lamina, the interlaminar space, and the inferior margin of the L4 lamina. The intersection of the medial fringe of the L5 superior articular process and the L5 lamina superior border, an important anatomical marker called the point L, could be visualized through the endoscope. Next, the endoscopic drill was used to grind the outer sphere of the lamina and facet joint to make them thinner. Next, the Kerrison rongeur was placed in the space between the lamina, and a portion of the lamina and facet joint was resected to reveal the yellow ligament. Then, ligamentum flavum was removed using endoscopic graspers to expose the dural sac and nerve root. On pulling the nerve using a nerve retractor, the nucleus pulposus tissue was revealed, which was then resected. After the disk was removed, the intervertebral space was cleared. The cartilage tissue on the superior and inferior end plates was scraped with a curette and reamer, and bony bleeding sites were observed. Endoscopic bone grafting was performed and an appropriately sized cage was implanted under C-arm guidance. Again, we confirmed using endoscopy that the nerve root and the dural sac pulsed well without nucleus pulposus remaining. Fourth, four pedicle screws and two connecting rods were inserted under fluoroscopic view. Finally, the surgical site was thoroughly rinsed with a large volume of normal saline, and the incision was sutured after sufficient hemostasis was obtained. The incision was covered with a sterile dressing and the operation was concluded (Fig. 4).

### Postoperative management

The postoperative management of both groups included electrocardiography, oxygen, bed rest, and conventional rehydration. Postoperatively, all patients were treated with parecoxib sodium as an anti-inflammatory analgesic, second-generation cephalosporins for infection prevention, and dexamethasone sodium phosphate injections for 3 days for nerve root edema. In the first postoperative week, the VAS and ODI of patients were evaluated, and operative and fluoroscopy times were noted. The occurrence of adverse events was also recorded. In the 1st month and 1st year postoperatively, the patients returned to the outpatient department for a follow-up visit and were assessed for VAS and ODI. During the follow-up visits, the occurrence of adverse events was continued to be recorded.

### Study variables

- Operative time and surgical complications
- VAS—The visual analogue scale is an instrument used to measure pain believed to range across a continuum of values, which cannot easily be measured directly. It consists of a straight horizontal line of fixed length, usually 100 mm. The ends are defined as the extreme limits of the pain. It is oriented from the left (worst) to the right (best) [20].
- ODI—It is a patient-completed questionnaire that gives a subjective percentage score for the level of function (disability) for activities of daily living in those rehabilitating from low back pain. There are six statements scored from 0 to 5, with the first statement scoring 0 through to the last scoring 5. All scores are then summed up for analysis [21].
- Radiological outcomes: The fusion rate, screw loosening, and cage subsidence were assessed. For determining fusion grades, each observer classified a patient into definite fusion (grade I), non-union (III, IV), or probable fusion (II) using Bridwell’s fusion grading system [22].

### Statistical analysis

For statistical analyses, IBM SPSS version 25.0 (IBM Corp., Armonk, NY, USA) was used. When measurement data were represented by the mean and standard deviation ($\bar{x}\pm s$), the *F* test was initially used to check for consistency of the variance, followed by a *t* test to compare the preoperative and postoperative 1-week, 1-month, and 1-year variables between the groups. A paired *t* test was selected for intra-group comparisons. When the measurement data were expressed as a percentage [*n* (%)], a Chi-square test was used to compare differences between groups. *P* values < 0.05 were considered to be statistically significant.

## Results

Preoperative age, VAS, and ODI of the two groups were compared, and no significant differences were obtained (*P* > 0.05) (Table 1).

**Table 1:** Comparison of preoperative data between two groups

	BLIF group	ULIF group	*P*
Age	50.83 ± 6.11	51.20 ± 6.49	0.822
VAS pro	7.40 ± 0.50	7.43 ± 0.50	0.790
ODI pro	43.17 ± 1.95	43.10 ± 2.11	0.934

All surgeries were successfully completed. The BLIF and ULIF groups differed in many aspects. For the former, the mean operative time was 98.07 ± 4.65 min, while that for the ULIF group was 134.53 ± 7.36 min, indicating that the mean operative time of the BLIF group was significantly shorter than that of the ULIF group (*P* < 0.05) (Table 2). With respect to complications, two cases of nerve root injury occurred in the BLIF group. In the ULIF group, leakage of cerebrospinal fluid occurred in one patient postoperatively (Table 3). Given prolonged dexamethasone treatment, the condition of the patients with nerve root injury got improved. The patients with their cerebrospinal fluid leaked had the incisions re-sutured and compression dressed. All three complications were well managed before discharge (*P* > 0.05).

**Table 2:** Comparison of operation time between two groups

Group	BLIF group	ULIF group	*P*
Operation time	98.07 ± 4.65	134.53 ± 7.36	< 0.001

**Table 3:** Comparison of the incidence of complication between two groups

Complication/Group	BLIF group	ULIF group	*P*
Nerve root injury	2	0	0.492
Leakage of cerebrospinal fluid	0	1	0.998

The VAS in the BLIF group decreased from preoperative 7.40 ± 0.50 to 3.23 ± 0.82 in the 1st postoperative week, 2.90 ± 0.40 in the 1st month, and 2.73 ± 0.45 in the 1st year (all *P* < 0.05). The VAS of the ULIF group decreased from preoperative 7.43 ± 0.50–3.20 ± 0.48 in the 1st postoperative week, 2.97 ± 0.41 in the 1st month, and 2.8 ± 0.41 in the 1st year (all *P* < 0.05). This indicated that in both BLIF group and ULIF groups, the postoperative VAS statistically significantly declined compared to the preoperative level (*P* < 0.05). In addition, effective pain relief could be maintained or even improved in the three postoperative follow-ups. Moreover, the comparison of preoperative and postoperative VAS showed no statistically significant difference between two groups (*P* > 0.05) (Tables 4 and 5).

**Table 4:** Comparison of VAS between two groups

Time/Group	BLIF group	ULIF group	*P*
Pro	7.40 ± 0.50	7.43 ± 0.50	0.795
Po 1st day	3.23 ± 0.82	3.20 ± 0.48	0.769
Po 1st month	2.90 ± 0.40	2.97 ± 0.41	0.530
Po 1st year	2.73 ± 0.45	2.80 ± 0.41	0.545

**Table 5:** Intra-group comparison of VAS of two groups

Group	Time	VAS	Time	VAS	*P*
BLIF group	Preoperation	7.40 ± 0.50	Postoperative 1st week	3.23 ± 0.82	0.000
	Preoperation	7.40 ± 0.50	Postoperative 1st month	2.90 ± 0.40	0.000
	Preoperation	7.40 ± 0.50	Postoperative 1st year	2.73 ± 0.45	0.000
	Postoperative 1st week	3.23 ± 0.82	Postoperative 1st month	2.90 ± 0.40	0.156
	Postoperative 1st week	3.23 ± 0.82	Postoperative 1st year	2.73 ± 0.45	0.004
	Postoperative 1st month	2.90 ± 0.40	Postoperative 1st year	2.73 ± 0.45	0.184
ULIF group	Preoperation	7.43 ± 0.50	Postoperative 1st week	3.20 ± 0.48	0.000
	Preoperation	7.43 ± 0.50	Postoperative 1st month	2.97 ± 0.41	0.000
	Preoperation	7.43 ± 0.50	Postoperative 1st year	2.80 ± 0.41	0.000
	Postoperative 1st week	3.20 ± 0.48	Postoperative 1st month	2.97 ± 0.41	0.005
	Postoperative 1st week	3.20 ± 0.48	Postoperative 1st year	2.80 ± 0.41	0.000
	Postoperative 1st month	2.97 ± 0.41	Postoperative 1st year	2.80 ± 0.41	0.026

As for the ODI, the scores of the BLIF group declined from preoperative 43.17 ± 1.95–6.23 ± 1.63 in the 1st postoperative week, 5.80 ± 0.96 in the 1st month, and 5.70 ± 0.92 in the 1st year. Meanwhile in the ULIF group, the score dropped from preoperative 43.10 ± 2.11–6.03 ± 0.89 in the 1st postoperative week, 5.77 ± 0.86 in the 1st month, and 5.63 ± 0.81 in the 1st year. The above data indicated that in both the BLIF and ULIF groups, the postoperative ODI improved significantly compared to the preoperative levels (*P* < 0.05). Furthermore, lumbar function was effectively improved and maintained in both groups, as shown from the fact that the ODI could be maintained or dropped further in the three postoperative follow-ups. Moreover, there was no significant difference in the ODI measured at each time point between the two groups (*P* > 0.05) (Tables 6 and 7).

**Table 6:** Comparison of ODI between two groups

Time/Group	BLIF group	ULIF group	*P*
Pro	43.17 ± 1.95	43.10 ± 2.11	0.934
Po 1st day	6.23 ± 1.63	6.03 ± 0.89	0.988
Po 1st month	5.80 ± 0.96	5.77 ± 0.86	0.833
Po 1st year	5.70 ± 0.92	5.63 ± 0.81	0.794

**Table 7:** Intra-group comparison of ODI of two groups

Group	Time	ODI	Time	ODI	*P*
BLIF group	Preoperation	43.17 ± 1.95	Postoperative 1st week	6.23 ± 1.63	0.000
	Preoperation	43.17 ± 1.95	Postoperative 1st month	5.80 ± 0.96	0.000
	Preoperation	43.17 ± 1.95	Postoperative 1st year	5.70 ± 0.92	0.000
	Postoperative 1st week	6.23 ± 1.63	Postoperative 1st month	5.80 ± 0.96	0.062
	Postoperative 1st week	6.23 ± 1.63	Postoperative 1st year	5.70 ± 0.92	0.023
	Postoperative 1st month	5.80 ± 0.96	Postoperative 1st year	5.70 ± 0.92	0.083
ULIF group	Preoperation	43.10 ± 2.11	Postoperative 1st week	6.03 ± 0.89	0.000
	Preoperation	43.10 ± 2.11	Postoperative 1st month	5.77 ± 0.86	0.000
	Preoperation	43.10 ± 2.11	Postoperative 1st year	5.63 ± 0.81	0.000
	Postoperative 1st week	6.03 ± 0.89	Postoperative 1st month	5.77 ± 0.86	0.008
	Postoperative 1st week	6.03 ± 0.89	Postoperative 1st year	5.63 ± 0.81	0.000
	Postoperative 1st month	5.77 ± 0.86	Postoperative 1st year	5.63 ± 0.81	0.043

**Fig. 5:**
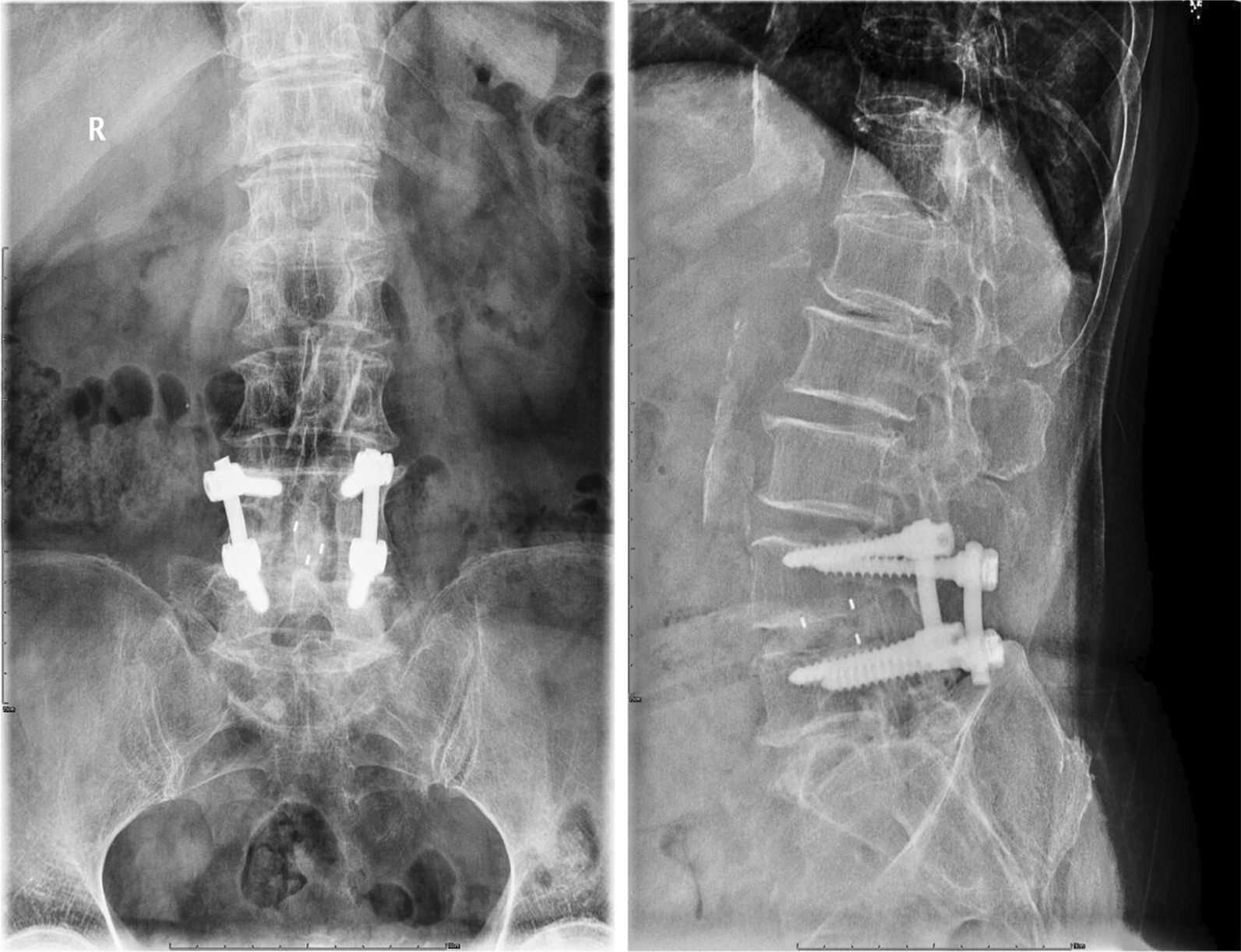
One of the “definite fusion” cases

**Fig. 6:**
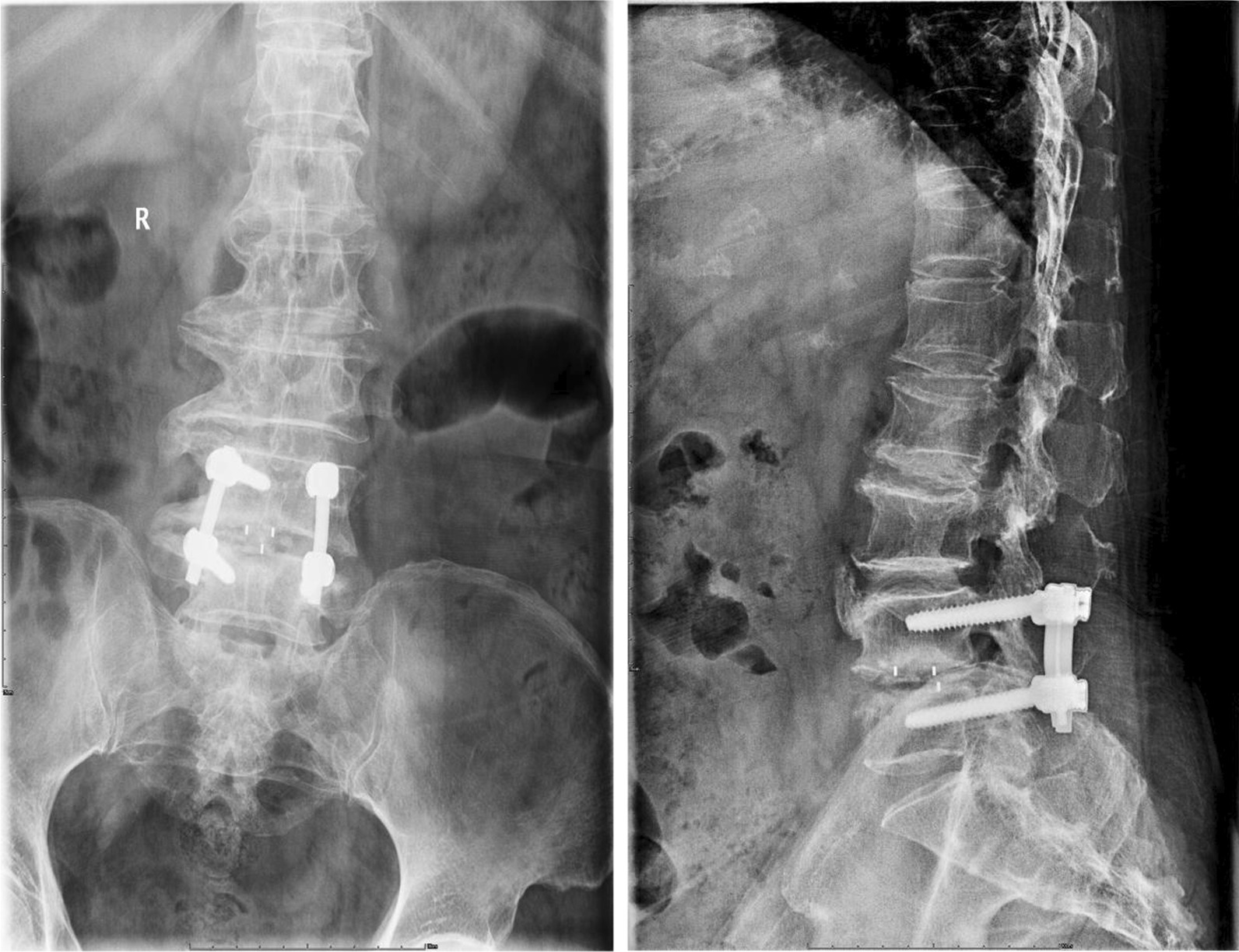
One of the “probable fusion” cases

**Fig. 7:**
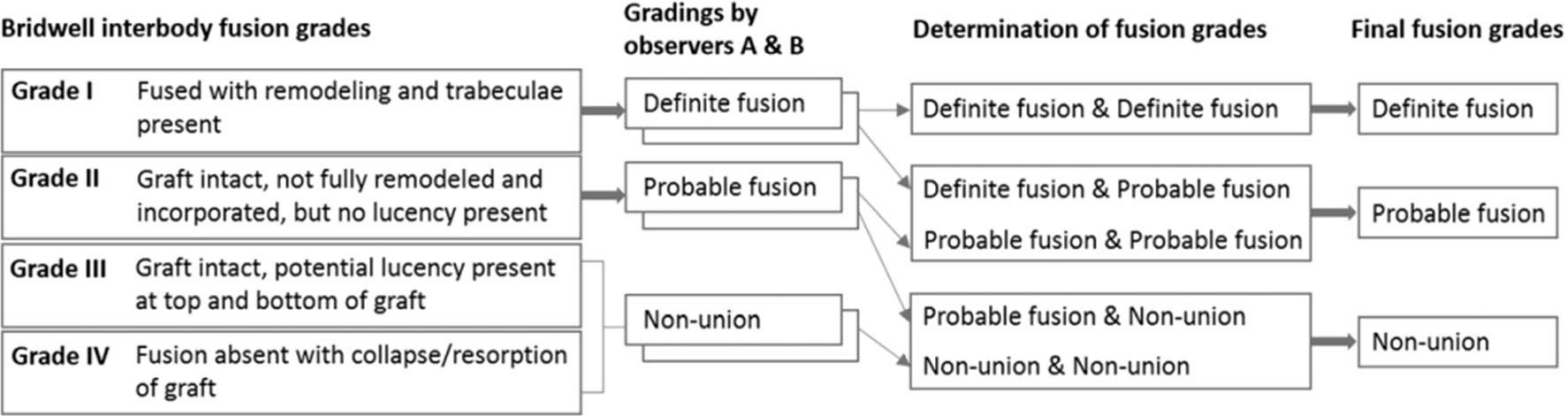
Two-step grading using two observers’ assessments of radiographs with Bridwell interbody fusion grading criteria to determine final fusion grades

In determining fusion grades, each observer classified a case with three grades as definite fusion (grade I) (Fig. 5), non-union (III, IV), or probable fusion (II) (Fig. 6) using Bridwell’s fusion grading system [12]. A case with a definite finding was determined by the agreements of two observers. A case with a probable finding was determined by the agreement of two observers or by one observer’s decision of definite finding with another’s decision of probable (Fig. 7). The fusion rates with definite (86.7%) and probable (6.7%) grades in the BLIF group were higher than that in the ULIF group (definite: 70% and probable: 3.3%), with a statistically significant difference (*P* < 0.05) (Table 8). One case of cage subsidence with no screw loosening occurred in each group; thus, there was no obvious difference between the two groups (*P* > 0.05) (Table 9).

**Table 8:** The comparison of fusion rate between two groups

Fusion rate/Group	*n*	Definite	Probable	Failure	*x*^2^	*P*
BLIF Group	30	26 (86.7)	2 (6.7)	2 (6.7)	4.465	0.107
ULIF Group	30	21 (70.0)	1 (3.3)	8 (26.7)

**Table 9:** Comparison of endoplant translocation between two groups

Radiological result/Group	BLIF Group (%)	ULIF Group (%)	*P*
Cage subsidence	3.33	3.33	1

## Discussion

In recent years, unilateral biportal spinal endoscopic surgery has gradually revived. South Korean scholars have especially made great contributions to this field [23–25], elevating the procedure from pure decompression to endoscopic fusion, and accelerating the development of this technology worldwide.

At present, biportal endoscopic spinal interbody fusion reported in the literature mostly represents transforaminal lumbar interbody fusion (TLIF) [23, 26, 27], while some authors also adopt posterior lumbar interbody fusion (PLIF).

During BLIF in this study, unilateral or bilateral spinal canal decompression could be performed through the interlaminar approach under full-endoscopic view: One facet joint was removed to preserve the lateral bone wall of the superior facet to protect the nerve root outlet from injury, and the resected bone was collected for subsequent autologous bone grafting. The space between the traversing root and the exiting roots could be noticed clearly, and the ligamentum flavum was cut laterally from the center to expose the dural sac, nerve roots, and other tissues in the spinal canal. Nucleus pulposus forceps, curettes, and reamers were used to remove the nucleus pulposus and strip the cartilage endplate tissue. Further, the endoscope could be extended into the intervertebral space for exploration to ensure that the cartilage endplate was fully scraped and the bone endplate was exposed.

**Fig. 8:**
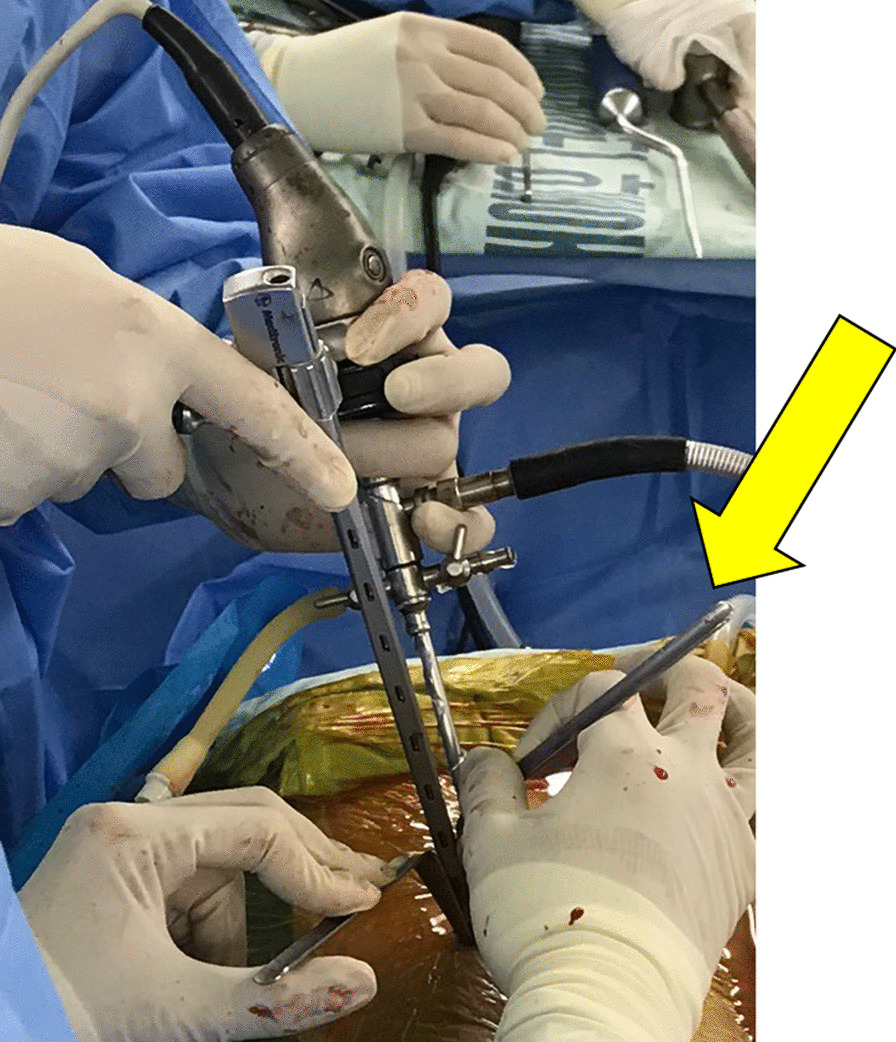
A specially designed retractor (yellow arrow) is used to fully expose the incision and protect the nerve root

Notably, a special channel could be placed for intervertebral bone grafting and a specially designed retractor was used to fully expose the incision and protect the nerve root (Fig. 8). Thus, the cage could be implanted into the intervertebral space with the assistance of endoscopy and fluoroscopy, which is undoubtedly an advantage over uniportal endoscopy [27]. The internal fixation method is similar to that of minimally invasive TLIF (MIS-TLIF) and is done using a percutaneous pedicle screw system with fluoroscopy-guided screw placement. In BLIF, percutaneous screws can be placed using the original channel.

In the realm of uniportal endoscopic fusion, Chinese peers have put in great efforts. Wu et al. [28] in a retrospective study compared open TLIF and endoscopic TLIF (Endo-TLIF) for VAS and ODI. They concluded that full-endoscopic TLIF is feasible for the treatment of single-segment lumbar degenerative diseases and is characterized by less trauma, quick recovery, and low cost.

A meta-analysis [29] compared Endo-LIF and MIS-TLIF. Based on the evidence generated by their study, there was no significant difference in the clinical efficacy and safety between Endo-LIF and MIS-TLIF for the treatment of lumbar degenerative diseases. Although Endo-LIF had a longer operative time, it had the advantages of lesser tissue trauma and rapid recovery after operation.

At present, Endo-LIF and MIS-TLIF are the mainstream and classic techniques of lumbar endoscopic fusion surgery. However, comparisons between biportal endoscopic lumbar interbody fusion and uniportal endoscopic lumbar interbody fusion have rarely been reported.

In this study, a retrospective analysis was conducted to compare the operative time, complications, VAS, ODI, and radiological outcomes between biportal endoscopic fusion and uniportal endoscopic fusion. There was no significant difference in VAS and ODI between the two procedures. Compared with uniportal endoscopic fusion surgery, biportal endoscopic fusion surgery takes lesser time and has a higher fusion rate. In the author’s opinion, this could be mainly attributed to the fact that in the biportal endoscopic technique, 30° endoscopy enables a wider surgical field of vision, greater maneuverability of instruments, and thus a broader, more thorough, and more efficient decompression range. In addition, compared with the uniportal alternative, the cage can be placed and transversely under biportal endoscopic visualization, which makes the placement of the cage more consistent with the mechanical effect. This is perhaps the reason for the higher fusion rate. At the same time, we also believe that biportal endoscopic fusion surgery, which does not rely on specialized instruments but uses traditional open surgical ones, is associated with a better learning curve than the uniportal endoscopic technique.

## Conclusion

Both BLIF and ULIF are effective and safe. These two techniques can effectively relieve pain and improve lumbar function, and there is no significant difference between the two groups.

Compared with the ULIF, BLIF offers advantages such as shorter operative time, higher fusion rate, and a better learning curve.

## Abbreviations

TESSYS: Transforaminal Endoscopic Spine System
YESS: Yeung Endoscopic Spine Surgery
MRI: Magnetic resonance imaging
VAS: Visual analogue scale
ODI: Oswestry Disability Index
cm: Centimeter
BLIF: Biportal endoscopic lumbar interbody fusion
ULIF: Uniportal endoscopic lumbar interbody fusion
